# An Acute Abdomen Mimicking Appendicitis

**DOI:** 10.5334/jbr-btr.1009

**Published:** 2016-01-29

**Authors:** Sokol Malasi, Redouane Kadi, Frederic Haven, Peter Matthys

**Affiliations:** 1 UZ Brussel, BE; 2 Cliniquesdeleurope, BE

**Keywords:** Appendix, Mucocele, Pseudomyxoma, CT

A 58-year-old-man presented to the emergency unit with abdominal pain and fever for three days. He had no history of nausea or vomiting. The abdominal examination revealed a minimally distended abdomen with tenderness in the right lower quadrant (RLQ). At palpation, there was no rebound tenderness or defence. Laboratory findings revealed inflammation and a high white blood cell count, predominantly neutrophils.

Contrast enhanced CT examination of the abdomen shows a 12cm, oval-shaped, low-attenuation cystic lesion (21 HU) with mural curvilinear calcifications (Figure 1, AM), contiguous with the distal appendix in the RLQ (Figure 2, A). A breach (Figure 1, arrow) is observed in the anterior aspect of the wall as well as an adjacent small fluid-like collection (Figure 1, asterisk) with no significant difference in density between this feature and the principal mass (19 HU). Moderate periappendiceal fat stranding is also seen (Figure 1, arrowhead).

**Figure 1:**
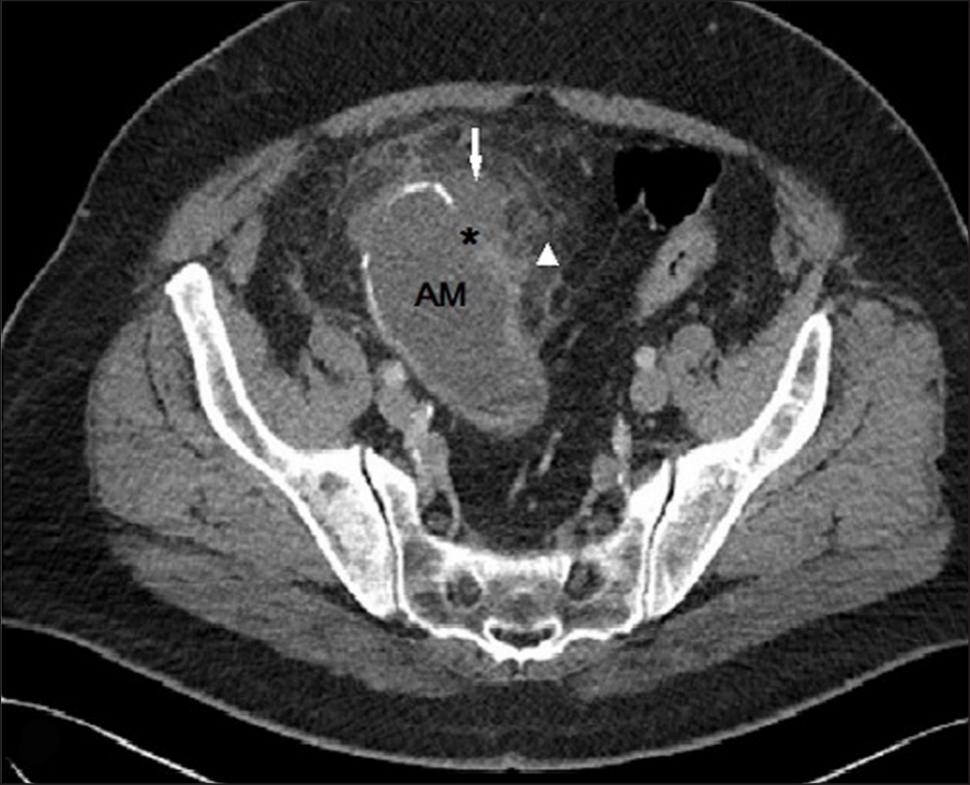


**Figure 2:**
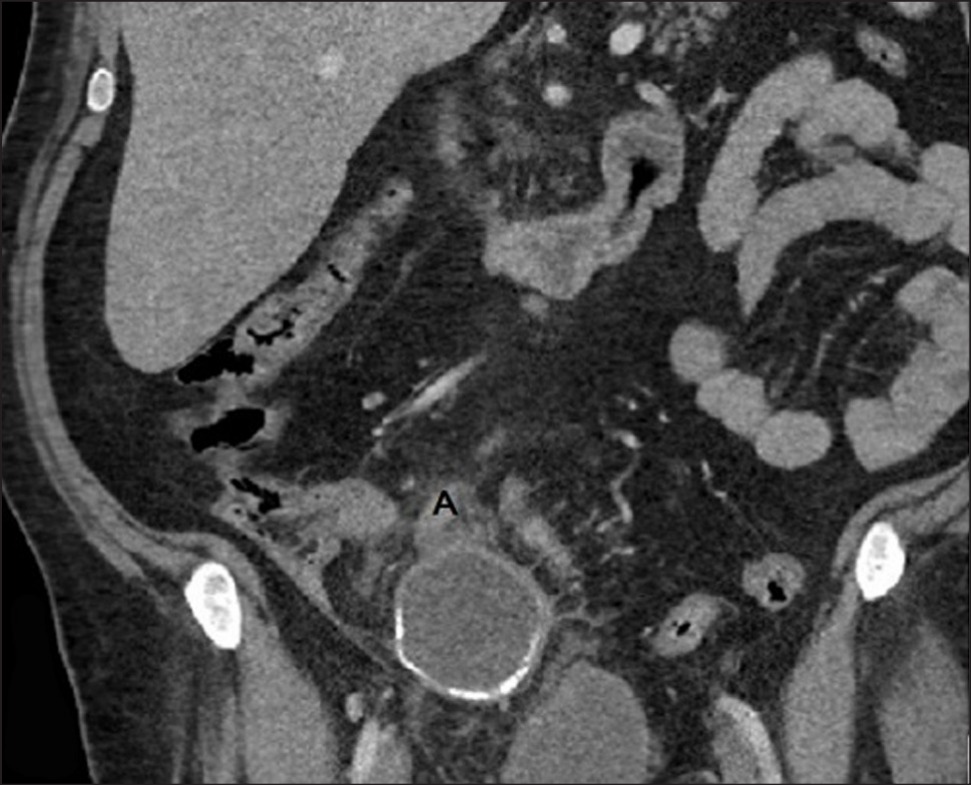


Based on these findings the diagnosis of a probably ruptured appendiceal mucocele (AM) was made. Surgery revealed the presence of greyish white gelatinous material into the peritoneal cavity confirming the diagnosis of rupture of a mucocele. Open laparotomy was chosen in order to minimize trauma and avoid further spilling of mucus. The gelatinous material was removed and the mucocele of the appendix was resected.

## Comment

AM is a progressive dilatation of the appendix from intraluminal accumulation of mucoid substance [1]. It may be a benign or malignant process. There are four histological types, including a simple mucocele or retention cyst and mucoceles associated with mucosal hyperplasia, mucinous cystadenoma and cystadenocarcinoma of the appendix. The worst complication of AM is pseudomyxoma peritonei (PMP), which is characterized by peritoneal dissemination and implantation of mucin-producing epithelial cells on the peritoneum and mucus accumulation within the peritoneal cavity, often leading to adhesions, fistula formations and intestinal obstruction. In such a case percutaneous aspiration biopsy is contraindicated. Therefore, open laparotomy is preferred to laparoscopic approaches in order to reduce the possibility of iatrogenic rupture and peritoneal contamination during resection [1].

PMP is difficult to treat and carries an uncertain prognosis, whether the mucocele originates from benign or malignant disease. CT is considered as the most accurate method of diagnostics and can be used to discover the signs specific to mucocele with a high accuracy (i.e., appendix lumen more than 1.3cm in size, cystic dilatation, relation with the base of the cecum and wall calcifications).

In our case, the histopathological examination confirmed the diagnosis of low-grade appendiceal mucinous neoplasm (mucinous cystadenoma). A specimen of the intra peritoneal mucus showed no evidence for presence of epithelial cells. The patient was referred to a specialised centre to perform further follow-up.

Although a mucocele is encountered in less than 0.5% of patients undergoing appendectomy, accurate preoperative diagnosis is important as it may reduce the possibility of rupture and peritoneal contamination during resection. It is the responsibility of the radiologist to alert the surgeon on the presence of such an entity before surgery.

## Competing Interests

The authors declare that they have no competing interests.

## References

[1] Dhage-Ivatury S, Sugarbaker PH (2006). Update on the surgical approach to mucocele of the appendix. J Am Coll Surg.

